# The Epidemiological Significance and Temporal Stability of Mycobacterial Interspersed Repetitive Units-Variable Number of Tandem Repeats-Based Method Applied to *Mycobacterium tuberculosis* in China

**DOI:** 10.3390/ijerph15040782

**Published:** 2018-04-17

**Authors:** Yang Li, Yi Hu, Mikael Mansjö, Qi Zhao, Weili Jiang, Solomon Ghebremichael, Sven Hoffner, Biao Xu

**Affiliations:** 1 Department of Epidemiology, China and Key Laboratory of Public Health Safety (Fudan University), School of Public Health, Fudan University, Ministry of Education, Shanghai 200032, China; yangli_fudan@163.com (Y.L.); zhaoqi@shmu.edu.cn (Q.Z.); wljiang@fudan.edu.cn (W.J.); bxu@shmu.edu.cn (B.X.); 2 Department of Public Health Sciences, Karolinska Institutet, SE-171 77 Stockholm, Sweden; sven.hoffner@ki.se (S.F.); biao.xu.1@ki.se (B.X); 3 The Public Health Agency of Sweden, SE-171 82 Solna, Sweden; mikael.mansjo@folkhalsomyndigheten.se (M.M.); solomon.ghebremichael@folkhalsomyndigheten.se (S.G.)

**Keywords:** *Mycobacterium tuberculosis*, MIRU-VNTR, epidemiological significance, temporal stability, China

## Abstract

This study aimed to validate the epidemiological significance and temporal stability of Mycobacterial Interspersed Repetitive Units-Variable Number of Tandem Repeats (MIRU-VNTR) typing in a genetically and geographically diverse set of clinical isolates from patients diagnosed with pulmonary tuberculosis in China. Between 2010 and 2013, a total of 982 *Mycobacterium tuberculosis* isolates were collected from four population-based investigations in China. Apart from the currently applied 24-locus MIRU-VNTR, six additional hypervariable loci were analyzed in order to validate the MIRU-VNTR combinations in terms of their epidemiological links, clustering time span, and paired geographic distance. In vitro temporal stability was analyzed for both individual MIRU-VNTR loci, and for several combinations of loci. In the present study, four MIRU-VNTR combinations, including the hypervariable loci 3820, 3232, 2163a, and 4120, were evaluated. All of these combinations obtained a Hunter-Gaston discriminatory index (HGDI) value over 0.9900 with a reduced clustering proportion (from 32.0% to 25.6%). By comparing epidemiological links, clustering time span, and paired geographic distance, we found that the performances of the four MIRU-VNTR combinations were comparable to the insertion sequence 6110 restriction fragment length polymorphism (IS*6110*-RFLP), and significantly better than that of 24-locus MIRU-VNTR genotyping alone. The proportion of temporally stable loci ranged from 90.5% to 92.5% within the combined MIRU-VNTR genotyping, which is higher than IS*6110*-RFLP (85.4%). By adding four hypervariable loci to the standard 24-locus MIRU-VNTR genotyping, we obtained a high discriminatory power, stability and epidemiological significance. This algorithm could therefore be used to improve tuberculosis transmission surveillance and outbreak investigation in China.

## 1. Introduction

Tuberculosis (TB) remains an important threat to public health worldwide. There has been a steady decline in both the incidence and prevalence of TB in China from 2010 to 2016, however TB remains a leading infectious disease [1]. Furthermore, drug-resistant TB, especially multidrug-resistant (MDR)-TB, has made the control of TB in China even more difficult [2]. The world health organization (WHO) estimated that China’s incidence of TB in 2016 was 64 cases per 100,000 people, of which 8.2% were MDR-TB/rifampicin-resistant (RR) TB cases [3].

The molecular epidemiological investigation of *Mycobacterium tuberculosis (M. tuberculosis)* has significantly increased our understanding of TB epidemiology, and contributed to population-based control of tuberculosis. Molecular genotyping suggests the potential transmission link between TB patients, enabling the identification of outbreaks, and laboratorial cross contamination [4]. Sequencing, e.g., whole genome sequencing, is very promising, but cannot easily be applied in high-burden TB settings, and there is still a demand for alternative molecular typing techniques. The earlier canonical reference method, the insertion sequence 6110 restriction fragment length polymorphism (IS*6110*-RFLP), cannot be universally applied due to difficulties in comparing results across laboratories, and handling of the strains with either too low [5,6] or too high numbers of IS*6110* copies [7]. In addition, the Beijing family strain has been predominant in China, accounting for 63.97% of the strain according to the national TB survey in 2007. Spoligotyping can be used to identify, but not to subdivide, the Beijing family, such as modern Beijing family strains, which limits its usefulness in the Chinese setting. For these reasons, the Polymerase Chain Reaction (PCR) based Mycobacterial Interspersed Repetitive Unit-Variable Number of Tandem Repeats (MIRU-VNTR) method is preferable, given its turnaround time and the convenience of its implementation [8,9,10]. Furthermore, it has a discriminatory power comparable to that of IS*6110*-RFLP [11]. 

For epidemiological applications, a typing test must have the ability to quickly and reliably differentiate between related bacterial isolates [12]. Moreover, the genetic stability will determine the applicability of the technology for different epidemiological purposes. In previous studies, several additional MIRU-VNTR hypervariable loci have been applied together with the traditional 24-locus MIRU-VNTR to achieve a higher resolution [13,14,15]. However, the epidemiological significance and temporal stability of this combined MIRU-VNTR genotyping deserves further evaluation due to variations in different populations and settings. For these reasons, we aimed to validate the epidemiological significance and temporal stability of the combined MIRU-VNTR typing in a genetically and geographically diverse set of clinical isolates from TB patients registered between 2010 and 2013 in China. 

## 2. Materials and Methods

### 2.1. Study Population 

Eight counties from four diverse geographical areas of China were chosen as study sites, including two from the Zhejiang province (East), two from the Liaoning Province (North), two from the Sichuan Province (Midwest) and two from the Guangdong Province (South). All of the pulmonary TB patients registered at the TB designated health facilities between 2010 and 2013 were enrolled. Their socio-demographic and clinical information was collected from medical records and the national TB registry system. Meanwhile, sputum samples were collected for culture and microbiological testing. Study subjects were defined as TB patients with positive culture results, whose *M. tuberculosis* isolates were further grown on Löwenstein–Jensen (LJ) medium for up to 4 weeks. The DNA was extracted according to the CTAB (Cetyltrimethylammonium Ammonium Bromide) method [16,17]. Extracted DNA was then sent to the TB molecular laboratory of Fudan University’s School of Public Health in Shanghai for molecular genotyping.

The study protocol and informed consents were approved by the Institutional Review Board of the School of Public Health, Fudan University (IRB00002408). All patients included in this study provided written informed consent for the use of clinical samples.

### 2.2. MIRU-VNTR Loci Selection and PCR Amplification

Briefly, a set of 15 discriminatory MIRU-VNTR loci (424, 577, 580, 802, 960, 1644, 1955, 2163b, 2165, 2401, 2996, 3192, 3690, 4052, 4156), 9 ancillary loci (154, 2059, 2347, 2461, 2531, 2687, 3007, 3171, 4348) and 6 hypervariable loci (2163a, 3232, 4120, 1982, 3336, 3820) were analyzed in all studied isolates. PCR was performed as previously reported [18]. The PCR products were identified by electrophoresis in 1.5% agarose gel using a 100-bp and a 50-bp DNA ladder (TAKARA). The size of the PCR fragments was defined using the Quantity One software, version 4.6.2 (BIO-RAD Laboratories, Hercules, CA, USA). DNA from the *M. tuberculosis* reference strain H37Rv was used as a positive control to assure the quality of amplicon sizing. Meanwhile, sterile water was used as a negative control. The exact number of complete repeats present was assigned using a derived allele-repeat table corresponding to the size of the PCR product. A MIRU-VNTR cluster was defined as two or more samples having the identical number of repeats at each of the MIRU-VNTR loci.

### 2.3. IS6110-RFLP Genotyping

DNA fingerprinting was conducted as earlier described [19]. In short, 32P-labeled 245 bp IS*6110* fragment was applied as a probe. H37Rv strains and sterile water were used as the positive and negative controls, respectively. After hybridization with the DNA probe, IS*6110*-RFLP was identified by autoradiography. The RFLP type was assigned based on the IS*6110* pattern (number and position of bands in the autoradiography). A cluster was defined as *M. tuberculosis* isolates harboring the identical IS*6110*RFLP pattern. 

### 2.4. Evaluation of Temporal Stability

A total of 294 clinical isolates were randomly selected in order to evaluate the temporal stability of all 30 of the studied MIRU-VNTR loci. The investigation of clonal stability was performed by isolating all the colonies in a clone 8 times on LJ media at intervals of 2 to 3 months over 2 years. The copy number of the studied MIRU-VNTR loci, as well as their temporal stability, were determined for every subculture from each isolate.

### 2.5. Data Collection and Measurement

The subjects with clustered isolates were interviewed in terms of socio-demographic characteristics, clinical manifestation, predisposing risk factors, and evidence of contact with patients with active TB. The collected information also included occupational, social, and recreational activities. 

The epidemiological links were defined as “Confirmed” in cases of simultaneously staying or working in the same place or by family relationship, while epidemiological links were defined as “Potential” in cases of residing in the same or the neighboring village, albeit in the same township. If the subjects lived in different townships or lacked a common neighborhood, their epidemiological links were regarded as “Not confirmed”. Clustering time span was defined as the period between the diagnosis dates of the initial and final patients in the cluster. Among the patients with an identical genotype, a paired geographic distance was calculated according to the longitude and latitude of their residential addresses.

### 2.6. Evaluation of Genotyping Methods and Statistical Analysis

The genotyping results were entered and analyzed using the BioNumerics software, version 7.6 (Applied Maths, Kortrijk, Belgium). The clusters were defined using the Jaccard index. The *h* value was calculated to define the allelic diversity of each locus. The equation was *h* = 1 − $\sum x_{j}^{2}\left(\frac{n}{n-1}\right)$. The “Hunter-Gaston discrimination index” (HGI) was applied to define the discriminative power of different genotyping strategies for comparison. The equation was: *D* = 1 − $\frac{1}{N\left(N-1\right)}\overset{s}{\underset{j=1}{\sum }}X_{j}\left(x_{j}-1\right)$ [19]. The stability of copies in MIRU-VNTR loci over time was examined using a Generalized Estimating Equation (GEE), which considered the effect caused by relativity between samples, and can be applied for repeated measurements data. The stability of MIRU-VNTR combinations was analyzed using Kaplan-Meier survival analysis. An “event” was defined to occur when the number of repeats at each MIRU-VNTR locus was observed to change. The time at which the “event” occurred was defined to be the midpoint between the two sampling times of the studied isolates. The log rank χ^2^ test was used to assess the differences in survival curves between methods. The Cochran-Mantel-Haenszel test was applied for categorical variables, while analysis of variance (ANOVA) and a multiplicity test (LSD) were used for continuous variables. Significant level (α) was set at 0.05. Data analysis was run using SAS v9.3 packages (SAS Institute, Cary, NC, USA).

## 3. Results

### 3.1. Patient Characteristics 

Of the 1065 isolates collected within the study period, 48 non-tuberculosis mycobacterium isolates and three *M. bovis* isolates were excluded. Of the 1014 *M. tuberculosis* isolates, 982 had molecular information available for spoligotyping MIRU-VNTR and IS*6110*-RFLP, and were included in the data analysis. 

Patients from the four areas had a similar average age ranging from 45 to 48 years. The male to female ratio was approximately 7:3. A total of 252 (25.7%) patients had cavities on chest radiographs. Nearly sixty percent of them had smear positive TB. In addition, the socio-demographic and clinical characteristics were similar in the four areas (Table 1). By means of spoligotyping, isolates from the Beijing family accounted for 69.0% (*n* = 678).

### 3.2. Genetic Diversity of Each MIRU-VNTR Locus

The number of repeats of each MIRU-VNTR locus was shown in Table S1. The genetic heterogeneity of each MIRU-VNTR locus was estimated by calculating *h* values (Table S2) for the whole sample set, as well as for the for Beijing or non-Beijing families. The *h* value differed from 0.836 for locus 3820 to 0.020 for locus 3171, with the genetic diversity being similar in the four areas. The loci 2059, 2347, 2687, and 3171 were highly homogeneous within the Beijing family of *M. tuberculosis* (*h* < 0.1). Of the six hypervariable loci, a high allelic diversity (*h* > 0.7) was seen in loci 3820, 3232, 2163a, and 4120.

### 3.3. Establishment of MIRU-VNTR Combinations

To identify a MIRU-VNTR combination suitable for the Chinese setting, we combined the 24-locus MIRU-VNTR genotyping with the four hypervariable loci (3820, 3232, 2163a, and 4120). As shown in Table 2, the combination of the 24 MIRU-VNTR loci with four additional loci had a higher discriminatory power than the 24-locus MIRU-VNTR genotyping alone (HGDI > 0.9900). Additionally, 123 clusters were identified by 24-locus MIRU-VNTR genotyping including 405 isolates (Table 3). When comparing the four combined MIRU-VNTR genotyping, the number of clusters was reduced from 98 to 82, as was the clustering proportion (from 32.0% to 25.6%).

### 3.4. Evaluation of Different Genotyping Strategies.

**Figure 1:**
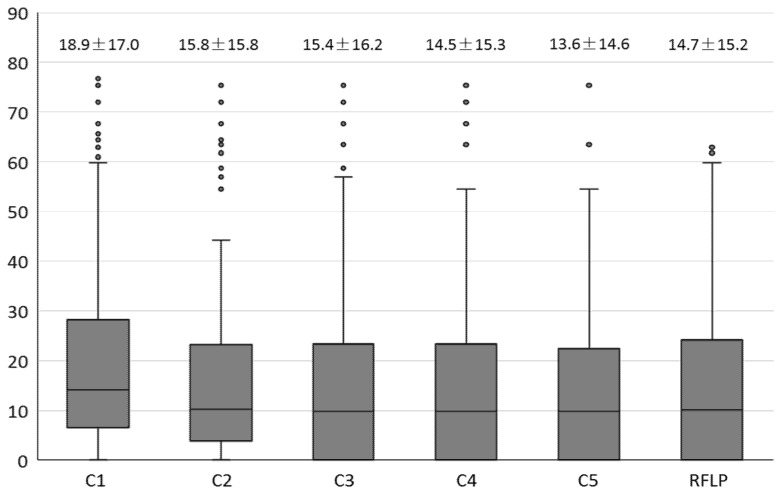
Paired geographic distance in different genotyping strategies.

We further assessed the epidemiological links, clustering time span, and paired geographic distance of the MIRU-VNTR combinations compared to 24-locus MIRU-VNTR genotyping and IS*6110*-RFLP. As shown in Table 4, it was observed that the MIRU-VNTR combination including four hypervariable loci identified more proven epidemiological links (27.9%) and potential epidemiological links (42.2%) compared to the 24-locus MIRU-VNTR genotyping alone (*p* < 0.001). Moreover, the proportion of clustering time span within 6 months increased significantly from 10.6% to 15.5% (*p* < 0.001). Among the paired strains with identical 24-locus MIRU-VNTR genotypes, the average geographic distance was 18.9 ± 17.0 km (Figure 1). With the combination including four additional loci, the paired distance was reduced to 13.6 ± 14.6 km (the corresponding value for IS*6110*-RFLP was 14.7 ± 15.2 km). 

Figure legend: In this figure, the distance between parents’ residence among pairs of clustered strains in different genotyping strategies was presented on the box plot, marked with the mean value and standard deviation. C1 refers to 24-locus MIRU VNTR alone; C2 refers to the combination of 24-locus MIRU VNTR plus locus 3820; C3 refers to the combination of 24-locus MIRU VNTR plus loci 3820 and 3232; C4 refers to the combination of 24-locus MIRU VNTR plus loci 3820, 3232, and 2163a; C5 refers to the combination of 24-locus MIRU VNTR plus loci 3820, 3232, 2163a, and 4120; RFLP refers to IS6110-RFLP; Multiplicity test (LSD) of ANOVA showed that the paired geographic distance of C2 to C5 was significantly shorter than that of C1 (*p* = 0.001~0.011), which was also comparable to the RFLP findings.

### 3.5. Temporal Stability of MIRU-VNTR Loci and Combinations

In the analysis of clonal isolates, pairs of strained clones of 294 randomly selected strains (including 202 and 92 isolates from the Beijing and non-Beijing families respectively) were analyzed (Table S3). With the exception of locus 2347, the 29 (96.7%) remaining MIRU-VNTR loci showed variant patterns differing by one to two copies of MIRU-VNTR over two years’ observation. Among these loci, 12 out of 29 MIRU-VNTR loci (41.4%) exhibited a variation in the number of repeat copies in one or two isolates within the first 12 months, while 17 out of 29 (58.6%) varied between the 12th and 24th months. Furthermore, the analysis based on the GEE model revealed a significant variation in the locus 3820 (*p* = 0.015), while the remaining loci were stable over the two years’ observation. 

**Figure 2:**
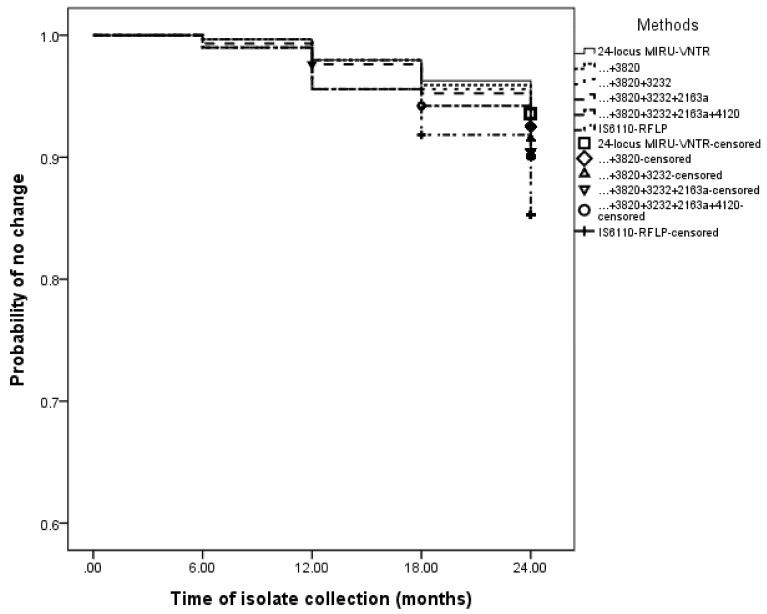
Evaluation of stability in different genotyping strategies applied on 294 randomly selected isolates.

As for the stability of MIRU-VNTR combinations, the Kaplan-Meier survival curves showed no separation of 24-locus MIRU-VNTR from other combinations over the two-year period (Figure 2). The proportion of temporally stable loci ranged from 90.5% to 92.5% within the four MIRU-VNTR combinations, which was comparable to 24 MIRU-VNTR (93.5%). IS*6110*-RFLP was the least stable, and the probability of no change at 24 months was 85.4%. 

## 4. Discussion

MIRU-VNTR has proven its capacity for typing *M. tuberculosis* isolates in terms of discriminatory power and convenience. However, the epidemiological implication of MIRU-VNTR on the transmission of TB remains to be empirically evaluated [8,20]. Furthermore, the 24-locus MIRU-VNTR strategy needs to be evaluated in order to determine whether it is sufficiently stable and discriminative to allow meaningful epidemiological study. In this study, we showed that additional hypervariable MIRU-VNTR loci combined with 24-locus MIRU-VNTR not only provided a high discriminatory typing strategy, but also manifested temporal stability, and an improved epidemiological significance.

The heterogeneity of MIRU-VNTR loci has varied in previous studies [8,21,22]. These studies reported that loci 424, 802, 960, 1955, 3007, 3690, and 4348 had higher allelic diversity (*h* > 0.60). However, in this study, loci 424, 802, 960, 3690, and 4348 were moderately homogeneous (0.2 < *h* < 0.6), while loci 1955 and 3007 exhibited low allelic diversity (*h* = 0.144, 0.189). It is possible that the previous studies may have suffered from a problem of representativeness, due to the fact that the sample selection was restricted to only one setting [23]. Moreover, we found that the four hypervariable MIRU-VNTR loci with the highest allelic diversity were loci 3820, 3232, 2163a, and 4120 (*h* > 0.7), and these were further combined with the traditional 24-locus MIRU-VNTR to improve the typing resolution. However, this combination was inconsistent with Allix’s study of a 4-locus set (loci 1982, 3232, 3820, and 4120) [24], as well as Luo’s study of a 3-locus set (loci 3820, 3232 and 4120) [25]. It indicated the genetic diversity of hypervariable MIRU-VNTR loci varied in a specific setting, and the optimized MIRU-VNTR combination should be systematically evaluated further.

As previously reported, the inclusion of the most hypervariable MIRU-VNTR loci facilitates subdivision of the Beijing family, resulting in a significantly improved discriminatory power [8,25,26]. In this study, we found that the clustering proportion for the combination with all four hypervariable loci was reduced to 25.6%, which was comparable to that of IS*6110*-RFLP. Therefore, our data reveal that the addition of hypervariable MIRU-VNTR loci to the strategy currently in use could render the discriminative power equal to that of IS*6110*-RFLP typing. 

Most importantly, we analyzed the epidemiological significance of clustering data by correlating them to the contact tracing information. Compared with traditional 24-locus MIRU-VNTR, the proportion of isolates with confirmed transmission links increased from 13.1% to 27.9% when clustered by the modified MIRU-VNTR genotyping. Moreover, the modified assay also showed a shorter clustering time span and geographic distance, which might validate its capability to indicate recent transmission chains in targeted population-based epidemiological studies. 

A valid estimation of epidemiologically unrelated TB cases depends on the stability of the biomarker used for the genotyping. An additional aim of this study was to quantify the stability of the MIRU-VNTR combination, in comparison to the currently used genotyping methods, over a two year period, as proposed in previous studies [27,28]. In our observation, 12 loci displayed an altered copy number within the first year, and 17 loci varied between the 12th and 24th months. This result is in accordance with previous findings [29], which indicated that the average time needed for one copy number change in MIRU-VNTR genotyping was 18 months. Furthermore, the four MIRU-VNTR combinations remained stable among more than 90% of the isolates for up to two years, which was comparable to the currently used 24-locus MIRU-VNTR genotyping, and better than IS6110-RFLP typing. This suggests that the 24-locus MIRU-VNTR genotyping plus the additional hypervariable loci will estimate recent transmission in China more accurately and reliably than the 24-MIRU-VNTR alone.

Compared with whole genome sequencing (WGS), MIRU-VNTR typing has a lower discriminatory power [30], however the lower cost of its use facilitates its application and scale-up in most developing countries. Furthermore, previous reviews have also highlighted that the methodological variation in conducting and analyzing WGS data could lead to heterogeneous results that are difficult to compare [31]. Therefore, a genotyping method with a proper MIRU-VNTR combination might be more suitable for population-based molecular epidemiological investigations in developing countries.

## 5. Conclusions

This study evaluated multiple MIRU-VNTR loci in comparison to IS*6110*-RFLP in terms of temporal stability and epidemiological significance. The results of the combined MIRU-VNTR genotyping are well in line with those of IS*6110* RFLP, and exhibit a relative stability suitable for indicating recent transmission. This study could constitute a basis for further molecular investigation of *M. tuberculosis* in China. 

## Figures and Tables

**Table 1:** Clinical characteristics of patients with tuberculosis in different geographic areas of China.

Variate	Number of Patients (%)			
	East *n* = 385	North *n* = 211	South *n* = 169	West *n* = 217
Age (mean ± SD)	48 ± 19.0	45 ± 18.3	47 ± 19.3	48 ± 19.8
Male (Sex)	262 (68.1)	135 (64.0)	128 (75.7)	154 (71.0)
Cavity on CXR	109 (28.3)	50 (23.7)	38 (22.5)	55 (25.3)
Sputum smear positive	233 (60.5)	118 (55.9)	107 (63.3)	137 (63.1)

**Table 2:** The discriminative power of the different combinations of Mycobacterial Interspersed Repetitive Units-Variable Number of Tandem Repeats (MIRU-VNTR) loci.

MIRU-VNTR Loci Combination	Cumulative Hunter-Gaston Discriminatory Index (HGDI)		
	Total	Strain Family	
		Beijing	Non-Beijing
24-locus MIRU VNTR	0.9887	0.9828	0.9922
+3820	0.9909	0.9897	0.9931
+3820 + 3232	0.9922	0.9915	0.9943
+3820 + 3232 + 2163a	0.9937	0.9922	0.9959
+3820 + 3232 + 2163a + 4120	0.9954	0.9938	0.9961

**Table 3:** Comparison of technical validation in different genotyping strategies.

Method	No. of Pattern	No. of Unique Pattern	No. of Clustered Pattern (Isolates)	Clustering Proportion (%)	HGDI
24-locus MIRU VNTR	700	577	123 (405)	41.2	0.9854
+3820	766	668	98 (314)	32.0	0.9935
+3820 + 3232	786	697	89 (285)	29.0	0.9947
+3820 + 3232 + 2163a	795	716	79 (266)	27.1	0.9952
+3820 + 3232 + 2163a + 4120	813	731	82 (251)	25.6	0.9960
IS*6110*-RFLP	811	726	85 (256)	26.1	0.9958

**Table 4:** Comparison of epidemiological links and clustering time span in different genotyping strategies.

Method	Epidemiology Link (%)			*p*-Value ^a^	Time of Clustering Time Span (%)					*p*-Value ^a^
	Confirmed	Potential	Not Confirmed		<6 Months	6–12 Months	12–24 Months	24–36 Months	≥36 Months	
24-locus MIRU VNTR	53 (13.1)	89 (22.0)	263 (64.9)	<0.001	43 (10.6)	40 (9.9)	92 (22.7)	148 (36.5)	82 (20.2)	<0.001
+3820	69 (22.0)	110 (35.0)	135 (43.0)		43 (13.7)	49 (15.6)	84 (26.8)	100 (31.8)	38 (12.1)	
+3820 + 3232	69 (24.2)	108 (37.9)	108 (37.9)		41 (14.4)	48 (16.8)	74 (26.0)	89 (31.2)	33 (11.6)	
+3820 + 3232 + 2163a	67 (25.2)	106 (39.8)	93 (35.0)		39 (14.7)	47 (17.7)	67 (25.2)	81 (30.5)	32 (12.0)	
+3820 + 3232 + 2163a + 4120	70 (27.9)	106 (42.2)	75 (29.9)		39 (15.5)	43 (17.1)	68 (27.1)	72 (28.7)	29 (11.6)	
IS*6110*-RFLP	67 (26.2)	105 (41.0)	84 (32.8)		4 7 (18.4)	45 (17.6)	40 (15.6)	89 (34.8)	35 (13.7)	

^a^*p*-value was analyzed by Cochran-Mantel-Haenszel test.

## Supplementary Materials

The following are available online at http://www.mdpi.com/1660-4601/15/4/782/s1, Table S1: The number of repeats at each MIRU-VNTR loci in the studied *M. tuberculosis* isolates, Table S2: Genetic diversity (*h* values) of MIRU-VNTR loci in the studied *M. tuberculosis* isolates, Table S3: Temporal stability of MIRU-VNTR loci within 2 years’ in-vitro culture.
