# Supplementary material for: The Epidemiological Significance and Temporal Stability of Mycobacterial Interspersed Repetitive Units-Variable Number of Tandem Repeats-Based Method Applied to Mycobacterium tuberculosis in China
Source: Int J Environ Res Public Health. 2018 Apr 17;15(4):782. doi: 10.3390/ijerph15040782 (PMC5923824; doi:10.3390/ijerph15040782)
Supplement: Supplementary file 1 [file ijerph-15-00782-s001.pdf]

Supplementary Materials

Table S1. The number of repeats at each MIRU-VNTR loci in the studied *M tuberculosis* isolates.

VNTR locus	VNTR alias	Number of repeats																						
		0	1	2	3	4	5	6	7	8	9	10	11	12	13	14	15	16	17	18	19	20	21	22
424	Mtub04	0	5	9	114	123	711	18	1	0	1	0	0	0	0	0	0	0	0	0	0	0	0	0
577	ETRC	0	10	11	50	905	6	0	0	0	0	0	0	0	0	0	0	0	0	0	0	0	0	0
580	MIRU04	0	57	48	799	35	42	1	0	0	0	0	0	0	0	0	0	0	0	0	0	0	0	0
802	MIRU40	0	10	108	775	56	29	1	3	0	0	0	0	0	0	0	0	0	0	0	0	0	0	0
960	MIRU10	0	13	189	719	49	12	0	0	0	0	0	0	0	0	0	0	0	0	0	0	0	0	0
1644	MIRU16	0	24	80	838	39	1	0	0	0	0	0	0	0	0	0	0	0	0	0	0	0	0	0
1955	Mtub21	0	2	0	13	14	908	5	13	9	16	2	0	0	0	0	0	0	0	0	0	0	0	0
2163b	QUB11b	4	50	22	55	86	240	429	64	11	17	4	0	0	0	0	0	0	0	0	0	0	0	0
2165	ETRA	14	4	61	190	713	0	0	0	0	0	0	0	0	0	0	0	0	0	0	0	0	0	0
2401	Mtub30	0	0	159	13	804	3	2	0	0	1	0	0	0	0	0	0	0	0	0	0	0	0	0
2996	MIRU26	0	43	8	25	38	114	149	541	56	8	0	0	0	0	0	0	0	0	0	0	0	0	0
3192	MIRU31	0	2	25	176	57	701	20	1	0	0	0	0	0	0	0	0	0	0	0	0	0	0	0
3690	Mtub39	0	2	60	26	793	74	24	2	1	0	0	0	0	0	0	0	0	0	0	0	0	0	0
4052	QUB26	1	8	4	6	29	21	74	166	537	44	76	13	0	0	3	0	0	0	0	0	0	0	0
4156	QUB4156	3	10	17	796	87	64	2	1	2	0	0	0	0	0	0	0	0	0	0	0	0	0	0
154	MIRU02	0	47	925	9	0	0	0	0	0	1	0	0	0	0	0	0	0	0	0	0	0	0	0
2059	MIRU20	0	19	948	11	3	0	1	0	0	0	0	0	0	0	0	0	0	0	0	0	0	0	0
2347	Mtub29	0	0	10	13	956	3	0	0	0	0	0	0	0	0	0	0	0	0	0	0	0	0	0
2461	ETRB	1	105	860	15	1	0	0	0	0	0	0	0	0	0	0	0	0	0	0	0	0	0	0
2531	MIRU23	0	0	10	17	29	885	39	2	0	0	0	0	0	0	0	0	0	0	0	0	0	0	0
2687	MIRU24	1	943	35	2	1	0	0	0	0	0	0	0	0	0	0	0	0	0	0	0	0	0	0
3007	MIRU27	0	6	76	881	16	0	1	2	0	0	0	0	0	0	0	0	0	0	0	0	0	0	0
3171	Mtub34	0	1	1	972	7	1	0	0	0	0	0	0	0	0	0	0	0	0	0	0	0	0	0
4348	MIRU39	1	6	205	743	26	0	1	0	0	0	0	0	0	0	0	0	0	0	0	0	0	0	0
2163a	QUB11a	68	0	12	11	0	3	316	27	76	454	11	4	0	0	0	0	0	0	0	0	0	0	0
3232	Qub3232	0	0	0	0	0	0	28	0	0	0	31	11	260	130	263	0	0	259	0	0	0	0	0
4120	VNTR4120	1	40	2	362	14	269	86	44	37	38	81	3	2	2	1	0	0	0	0	0	0	0	0
1982	QUB18	8	66	8	0	4	33	114	66	84	508	63	28	0	0	0	0	0	0	0	0	0	0	0
3336	VNTR3336	0	0	0	0	764	109	109	0	0	0	0	0	0	0	0	0	0	0	0	0	0	0	0
3820	VNTR3820	46	0	8	0	72	7	77	7	20	23	15	19	17	120	88	314	101	0	22	7	4	4	11

Table S2. Genetic diversity (*h* values) of MIRU-VNTR loci in the studied *M tuberculosis* isolates.

Schemes of VNTR locus	VNTR locus	VNTR alias	Total	Strain family	
				Beijing	Non-Beijing
Discriminatory loci	424	Mtub04	0.447	0.424	0.535
	577	ETRC	0.148	0.150	0.140
	580	MIRU04	0.329	0.342	0.278
	802	MIRU40	0.361	0.329	0.482
	960	MIRU10	0.424	0.428	0.412
	1644	MIRU16	0.263	0.264	0.259
	1955	Mtub21	0.144	0.140	0.162
	2163b	QUB11b	0.634	0.625	0.672
	2165	ETRA	0.432	0.415	0.492
	2401	Mtub30	0.304	0.301	0.316
	2996	MIRU26	0.653	0.649	0.668
	3192	MIRU31	0.454	0.431	0.530
	3690	Mtub39	0.338	0.342	0.319
	4052	QUB26	0.658	0.670	0.605
	4156	QUB4156	0.331	0.344	0.272
Ancillary loci	154	MIRU02	0.110	0.108	0.120
	2059	MIRU20	0.068	0.062	0.091
	2347	Mtub29	0.052	0.059	0.021
	2461	ETRB	0.222	0.194	0.328
	2531	MIRU23	0.185	0.180	0.206
	2687	MIRU24	0.077	0.008	0.062
	3007	MIRU27	0.189	0.200	0.141
	3171	Mtub34	0.020	0.020	0.021
	4348	MIRU39	0.384	0.374	0.424
Hypervariable loci	2163a	QUB11a	0.732	0.723	0.756
	3232	Qub3232	0.752	0.753	0.731
	4120	VNTR4120	0.692	0.705	0.633
	1982	QUB18	0.589	0.607	0.543
	3336	VNTR3336	0.367	0.372	0.365
	3820	VNTR3820	0.836	0.832	0.855

Table S3. Temporal stability of MIRU-VNTR loci within 2 years 'in-vitro culture

VNTR locus	VNTR alias	baseline	6th month		12th month		18th month		24th month		P-value [a]
		Total	n	%	n	%	n	%	n	%	
424	Mtub04	294	0	0	1	0.34	1	0.34	2	0.68	0.176
577	ETRC	294	0	0	1	0.34	2	0.68	3	1.02	0.930
580	MIRU04	294	1	0.34	2	0.68	3	1.02	5	1.7	0.930
802	MIRU40	294	0	0	1	0.34	2	0.68	3	1.02	0.076
960	MIRU10	294	0	0	1	0.34	1	0.34	2	0.68	0.058
1644	MIRU16	294	1	0.34	3	1.02	4	1.36	5	1.7	0.176
1955	Mtub21	294	0	0	1	0.34	1	0.34	2	0.68	0.055
2163b	QUB11b	294	0	0	1	0.34	1	0.34	2	0.68	0.087
2165	ETRA	294	0	0	0	0	1	0.34	1	0.34	0.058
2401	Mtub30	294	0	0	0	0	1	0.34	3	1.02	0.176
2996	MIRU26	294	0	0	1	0.34	1	0.34	2	0.68	0.058
3192	MIRU31	294	0	0	0	0	1	0.34	1	0.34	0.052
3690	Mtub39	294	0	0	0	0	1	0.34	1	0.34	0.176
4052	QUB26	294	0	0	1	0.34	1	0.34	2	0.68	0.058
4156	QUB4156	294	1	0.34	1	0.34	1	0.34	3	1.02	0.058
154	MIRU02	294	0	0	0	0	1	0.34	2	0.68	0.930
2059	MIRU20	294	0	0	0	0	1	0.34	3	1.02	0.930
2347	Mtub29	294	0	0	0	0	0	0	0	0	0.930
2461	ETRB	294	0	0	0	0	1	0.34	2	0.68	0.176
2531	MIRU23	294	0	0	0	0	1	0.34	3	1.02	0.930
2687	MIRU24	294	0	0	0	0	1	0.34	2	0.68	0.930
3007	MIRU27	294	0	0	0	0	0	0	1	0.34	0.930
3171	Mtub34	294	0	0	0	0	0	0	1	0.34	0.930
4348	MIRU39	294	0	0	0	0	1	0.34	1	0.34	0.058
2163a	QUB11a	294	0	0	0	0	0	0	1	0.34	0.052
3232	Qub3232	294	0	0	0	0	1	0.34	1	0.34	0.058
4120	VNTR4120	294	0	0	0	0	0	0	1	0.34	0.176
1982	QUB18	294	0	0	0	0	1	0.34	1	0.34	0.058
3336	VNTR3336	294	0	0	0	0	0	0	1	0.34	0.176
3820	VNTR3820	294	1	0.34	2	0.68	3	1.02	5	1.7	0.015[b]

a P value was calculated by analyzing the time-effect in the Generalized Estimating Equations

b $p<0.05$
